# State of the Art in Idiopathic Pulmonary Fibrosis

**DOI:** 10.3390/cells11162487

**Published:** 2022-08-11

**Authors:** Elie El Agha, Malgorzata Wygrecka

**Affiliations:** 1 Department of Medicine II, Internal Medicine, Pulmonary and Critical Care, Universities of Giessen and Marburg Lung Center (UGMLC), Justus-Liebig University Giessen, German Center for Lung Research (DZL), 35392 Giessen, Germany; 2 Department of Medicine V, Internal Medicine, Infectious Diseases and Infection Control, Universities of Giessen and Marburg Lung Center (UGMLC), Justus-Liebig University Giessen, German Center for Lung Research (DZL), 35392 Giessen, Germany; 3 Cardio-Pulmonary Institute (CPI), Justus-Liebig University Giessen, 35392 Giessen, Germany; 4 Institute for Lung Health (ILH), Justus-Liebig University Giessen, 35392 Giessen, Germany

Idiopathic pulmonary fibrosis (IPF) is a form of usual interstitial pneumonia (UIP), though its origin is unknown. IPF remains one of the most aggressive and lethal forms of interstitial lung diseases. Extensive basic and clinical research during the past decade has uncovered critical aspects related to the pathophysiological events that lead to the formation of fibrotic scars in the lungs of IPF patients. Our current understanding of IPF pathogenesis includes repetitive/chronic injury to alveolar epithelial type 2 cells (AEC2) that triggers an aberrant wound healing response associated with the activation of fibroblasts and the infiltration of immune cells. This leads to the excessive accumulation of myofibroblasts that deposit extracellular matrix (ECM) proteins, particularly collagen and fibronectin. All aforementioned processes culminate in detrimental scarring of the lung tissue, loss of AEC2 and AEC1, and respiratory failure [1].

In this Special Issue, Wasnick et al. describe two subsets of AEC2 in both healthy and fibrotic lungs [2]. Using the lysosomal dye, LysoTracker, to stain lamellar bodies in AEC2, the authors demonstrate that *bona fide* AEC2 (Lyso^high^) is the predominant AEC2 subset in the healthy lung, and that this population is largely replaced by an intermediate AEC state (Lyso^low^) in bleomycin-induced pulmonary fibrosis and in human IPF. Lyso^low^ cells express, in addition to typical AEC2 markers, markers of basal cells, thus highlighting epithelial cell plasticity as a source of cellular heterogeneity in IPF [3,4,5,6,7]. Lyso^low^ cells are also reminiscent of the tdTomato-low (tdTom)^low^ cells, identified using the AEC2 lineage-tracing mouse line (*Sftpc^Cre-ERT2^; tdTomato^flox^*) [8,9,10]. Such tdTom^low^ cells have been shown to expand during compensatory lung growth following pneumonectomy and were therefore dubbed injury-activated alveolar progenitors (IAAPs) [8,9]. In this Special Issue, Lv et al. show that upon bleomycin injury in mice, IAAPs are amplified alongside a loss of mature AT2 cells [11]. They also show that the intratracheal instillation of recombinant fibroblast growth factor 10 (rFGF10) is both preventive and therapeutic against bleomycin-induced pulmonary fibrosis. These findings accord with previous data that utilized a transgenic approach to overexpress *Fgf10* [12]. Importantly, Lv et al. also demonstrate that therapeutic rFGF10 treatment further boosts IAAP expansion [11]. In the future, it will be worth comparing the colony-forming efficiency of human AT2 subsets and their mode of interaction with mesenchymal niche cells using organotypic co-culture models. In this context, Vazquez-Armendariz et al. provide a comprehensive review on various three-dimensional (3D) models that can be used to study cell–cell interactions [13]. These 3D models include hydrogels, precision-cut lung slices (PCLSs), lung organoids, and lung-on-chip devices. These approaches can be particularly useful in analyzing intermediate cell states that persist during fibrosis, and in evaluating molecular signaling pathways which drive lung tissue scarring. The ultimate goal of such approaches is to improve our understanding of the events that mediate the onset, progression, and resolution of lung fibrosis, and to identify novel therapeutic targets via high-throughput drug screening.

Despite the fact that IPF is considered as a “distal” lung disease, with a central role of AEC2 malfunction during its pathogenesis, Chakraborty et al. highlight recent developments that point towards the involvement of airway epithelial cells in IPF pathobiology [14]. The contribution of airway epithelial cells to the development of IPF is supported by multiple lines of evidence, including the cellular composition and morphological alterations of IPF airways, strong associations between the *MUC5B* promoter *rs35705950* polymorphism and the risk of IPF, and the bronchiolization of distal airspaces in IPF lungs. The contribution of airway epithelial cells to IPF pathogenesis is also addressed by Schramm et al., who discuss the role of the ErbB receptor–ligand system in lung fibrosis [15]. Intriguingly, the deregulation of the ErbB receptor-ligand axis is mainly observed in the regions of alveolar bronchiolization. This underlies the pivotal role of ErbB receptors in the reprogramming of airway epithelial cells, and thereby honeycomb cyst formation and IPF progression. The authors of this comprehensive review emphasize the dual role of ErbB receptors and their ligands in lung fibrosis and indicate the need to elaborately characterize the dynamics and causal flows in the ErbB signaling networks in acute versus chronic lung injury [15].

The cellular heterogeneity of IPF is further discussed by Preisendörfer et al., who stress the potential role of B cells in IPF pathobiology [16]. The pathological relevance of B-cell accumulation in the lungs of IPF patients is supported by the presence of circulating autoantibodies and the increased concentration of B-lymphocyte stimulator factor (BLyS) in IPF plasma. The authors build upon these findings and show increased levels of FK506-binding protein 11 (FKBP11), an antibody-folding catalyst, in IPF lungs. Mechanistically, they demonstrate that FKBP11 expression is elevated following the differentiation of B cells into antibody-secreting plasma cells and upon the induction of ER stress in A549 cells. The latter may lead to higher susceptibility of A549 cells to ER stress-induced cell death. Although the authors failed to demonstrate the importance of FKBP11 for antibody production in the loss-of-function approaches, these results provide further evidence for the role of auto-immunogenicity in IPF pathogenesis [16].

IPF is also associated with a set of metabolic disorders and targeting cellular metabolism, particularly in mesenchymal cells, represents an exciting therapeutic avenue [17,18,19,20,21]. Kheirollahi et al. provide a systematic gene expression analysis of insulin-like growth factor (IGF) signaling components during embryonic murine lung development, as well as bleomycin-induced pulmonary fibrosis in mice and human IPF [22]. The authors demonstrate significant upregulation of IGF1 and several IGF binding proteins (IGFBPs) in parallel with marked downregulation of IGF1 receptor (IGF1R) in lung fibrosis. They also address the impact of matrix stiffness on the fibroblast response to IGF1 treatment and the interconversion to either lipofibroblasts or myofibroblasts [22]. The lipofibroblast–myofibroblast interconversion was recently shown to be involved in the development and resolution of experimental lung fibrosis [21,23,24].

One of the factors that contribute to the incurable nature of IPF is the fact that patients are already in advanced disease stages at the time of diagnosis. As such, treatment mainly focuses on treating the symptoms and slowing down disease progression rather than treating early events that initiate downstream pathological signaling cascades. Accordingly, efforts that seek to find alternative therapeutic agents against IPF are still ongoing.

Current treatment modalities for IPF patients rely on two approved drugs: pirfenidone, which is believed to act as a transforming growth factor beta 1 and hedgehog signaling inhibitor [25], and nintedanib, which is a small molecule inhibitor of multiple receptor tyrosine kinases (RTKs). Although these two medications slightly prolong survival of IPF patients, they do not halt disease progression. Moreover, they possess a marked side-effect profile, which may lead to the discontinuation of antifibrotic therapy. In this Special Issue, Takehara et al. [26] retrospectively analyzed the discontinuation rates of pirfenidone and nintedanib in a cohort of 261 patients in Japan, 77 of which were excluded because either the antifibrotic agent was switched or the observation period was less than a year. Analysis of the remaining patient data showed that the discontinuation rate was around half within one year of treatment. Over the entire treatment period, the discontinuation rates were similar for the two drugs; however, discontinuation due to adverse events was higher for nintedanib, with diarrhea and liver dysfunction being the more common reasons for cessation. Discontinuation due to disease progression or hospital transfer was higher for pirfenidone than nintedanib. The authors associated the adverse effects of nintedanib with a lower body mass index (BMI) and recommended a reduced starting dose and closer attention paid to adverse events at the initiation of nintedanib treatment to improve tolerability, achieve longer treatment, and improve prognosis [26].

Korfei et al. contribute to the Special Issue with a comprehensive review on the current knowledge regarding IPF pathophysiology, the associated cellular and molecular mechanisms, clinical management, as well as novel therapeutic targets and therapies in development [27]. Importantly, the authors draw lessons about histone deacetylases (HDACs) from the cancer field and highlight the imbalance between increased HDAC activity in fibroblasts and bronchiolar basal cells, and decreased HDAC activity in AEC2 in IPF. Such an imbalance leads to fibroblast proliferation, ECM deposition and bronchiolar basal cell hyperproliferation from one side, and AEC2 endoplasmic reticulum (ER) stress, senescence, and apoptosis from the other side. All these processes ultimately culminate in fibrosis development and progression. Accordingly, the authors propose targeting HDACs as a novel therapeutic option for IPF [27].

Braubach et al. go beyond IPF and provide a broad characterization of fibroelastotic remodeling (FER), its anatomic distribution, and clinical association [28]. FER is observed in pleuroparenchymal fibroelastosis (PPFE), which can either be of idiopathic origin or linked to autoimmune disorders. All PPFE manifestations share a rather poor prognosis and a similar histological feature of the fibrous obliteration of alveolar airspaces. The authors stress the need of histological characterization of FER and its clear demarcation from other interstitial lung diseases, as this may provide the basis for studies addressing molecular mechanisms underlying PPFE development [28].

In conclusion, we present a collection of articles in this Special Issue that touch on all aspects of IPF, from the molecular mechanisms driving disease development and progression, to translational models enabling high-throughput drug screening, finishing on clinical studies evaluating adverse effect profiles of drugs approved for IPF treatment. Despite immense improvements in understanding the molecular basis of IPF, further research is urgently needed to pursue innovative translational studies in order to learn more about the enormous repertoire of molecular tricks that lung cells use to adapt to stress conditions and fight against damaging agents.
